# Survey of mosquito species and mosquito-borne viruses in residential areas along the Sino–Vietnam border in Yunnan Province in China

**DOI:** 10.3389/fmicb.2023.1105786

**Published:** 2023-02-23

**Authors:** Fengjuan Tian, Jimin He, Shanlin Shang, Zhongyan Chen, Yumei Tang, Man Lu, Changzhi Huang, Xiaofang Guo, Yigang Tong

**Affiliations:** ^1^Beijing Advanced Innovation Center for Soft Matter Science and Engineering, College of Life Science and Technology, Beijing University of Chemical Technology, Beijing, China; ^2^The Second Affiliated Hospital of Kunming Medical University, Kunming, Yunnan, China; ^3^Malipo County Center for Disease Control and Prevention, Wenshanzhou, Yunnan, China; ^4^Funing County Center for Disease Control and Prevention, Wenshanzhou, Yunnan, China; ^5^Yunnan Provincial Key Laboratory of Vector-borne Disease Control and Research, Yunnan Institute of Parasitic Diseases Control, Puer, Yunnan, China

**Keywords:** mosquito, arbovirus, virus isolation, Sino–Vietnam border, mosquito-borne viruses

## Abstract

Mosquitoes are capable of carrying complex pathogens, and their feeding habits on the mammalian blood can easily mediate the spread of viruses. Surveillance of mosquito-based arbovirus enables the early prevention and control of mosquito-borne arboviral diseases. The climate and geography of Yunnan Province in China are ideal for mosquitoes. Yunnan shares borders with several other countries; therefore, there exists a high risk of international transmission of mosquito-mediated infectious diseases. Previous studies have focused more on the Sino–Laos and Sino–Myanmar borders. Therefore, we focused on the neighborhoods of Malipo and Funing counties in Wenshan Prefecture, Yunnan Province, China, which are located along the Sino–Vietnam border, to investigate the species of mosquitoes and mosquito-borne viruses in the residential areas of this region. This study collected 10,800 mosquitoes from 29 species of 8 genera and grouped to isolate mosquito-borne viruses. In total, 62 isolates were isolated and classified into 11 viral categories. We demonstrated a new distribution of mosquito-borne viruses among mosquitoes in border areas, including Tembusu and Getah viruses, which can cause animal outbreaks. In addition, Dak Nong and Sarawak viruses originating from Vietnam and Malaysia, respectively, were identified for the first time in China, highlighting the complexity of mosquito-borne viruses in the Sino–Vietnam border region. The awareness of the importance of viral surveillance and prevention measures in border areas should be further encouraged to prevent future outbreaks of potentially infectious diseases.

## 1. Introduction

Mosquito-borne viruses are an important component of emerging infectious diseases and pose notably global public health risks. Increasing international exchange poses greater challenges for epidemic control in border areas. Yunnan Province in China has a subtropical monsoon climate and a complex and diverse environment rich in natural resources, which is suitable for many plants and animals, providing a habitat for mosquitoes to survive and breed. In addition, conditions are favorable for the spread of mosquito-borne viruses. Over the last half-century, mosquitoes belonging to more than 10 families have been identified in Yunnan Province. The abundance of species and the suitability of the environment for host viruses increase the risk of complex viruses circulating in this region. In addition, Yunnan Province has the highest abundance and most widely distributed density of mosquito-borne viruses in China compared to other provinces (Atoni et al., 2020). The southeastern part of Yunnan Province shares borders with several neighboring countries. Owing to the different living habits and medical conditions of people in neighboring countries, the prevalence of pathogens varies from country to country. Since mosquitoes can migrate long distances (Huestis et al., 2019), and due to the expanding distribution of mosquitoes affected by climate change (Watts et al., 2018), Yunnan is at high risk of mosquito-borne pathogen outbreaks imported from neighboring countries. For instance, in 2013, an unexpected large outbreak of dengue fever was reported in Xishuangbanna Dai Autonomous Prefecture in Yunnan Province, whose evolutionary analysis showed that this dengue virus may have originated from neighboring countries (Zhang et al., 2014). Therefore, the abundance, distribution, and pathogenicity of circulating mosquito-borne viruses in border areas deserve more in-depth attention in the context of the development and implementation of disease prevention and control strategies. However, previous studies on arboviruses in Yunnan have mainly focused on the Sino–Myanmar and Sino–Laos border areas (Wang et al., 2011; Feng et al., 2012; Zeng et al., 2018; Hameed et al., 2020; Fang et al., 2021), with fewer studies being conducted on the Sino–Vietnam border area (Zhou et al., 2009). The northern part of Vietnam, bordering Yunnan, is an endemic area for dengue fever and the Japanese encephalitis virus (JEV), and an outbreak of the Zika epidemic occurred there during 2016–2017 (Jakobsen et al., 2019; Nealon et al., 2019; Nguyen-Tien et al., 2019, 2021). People living in the areas around Sino–Vietnam border may be at an increased risk of cross-border infectious diseases. In this study, we conducted a survey of mosquito and arbovirus species in the residential areas of Malipo and Funing counties of Wenshan Prefecture, Sino–Vietnam border area in Yunnan from 2020 to 2021, to monitor and describe the distribution of vectors and arboviruses in this area.

## 2. Materials and methods

### 2.1. Survey of mosquito species and specimen collection

From 21st August to 5th September 2020, five townships in Malipo County, Wenshan Prefecture, namely Malipo, Donggan, Mangkou Yao, Babu, and Tianbao Townships, were selected according to their altitude and ecological environment. Mosquitoes were trapped in the livestock corrals during two nights in a row using a CDC Light Trap (John W. Hock Company, Gainesville, FL, United States). Mosquitoes were captured from the woods or bamboo forests near residential areas during the day using an electric mosquito catcher. Trapping personnel wore long clothes and pants and followed personal protective measures to avoid mosquito bites. In a survey of residential areas inside and outside of courtyards and households, and in surrounding bamboo forests and woods, we used the pipette method to collect mosquito larvae (pupae) in containers of stagnant water, reared them until eclosion, and then collected the adult mosquitoes.

**Figure 1:**
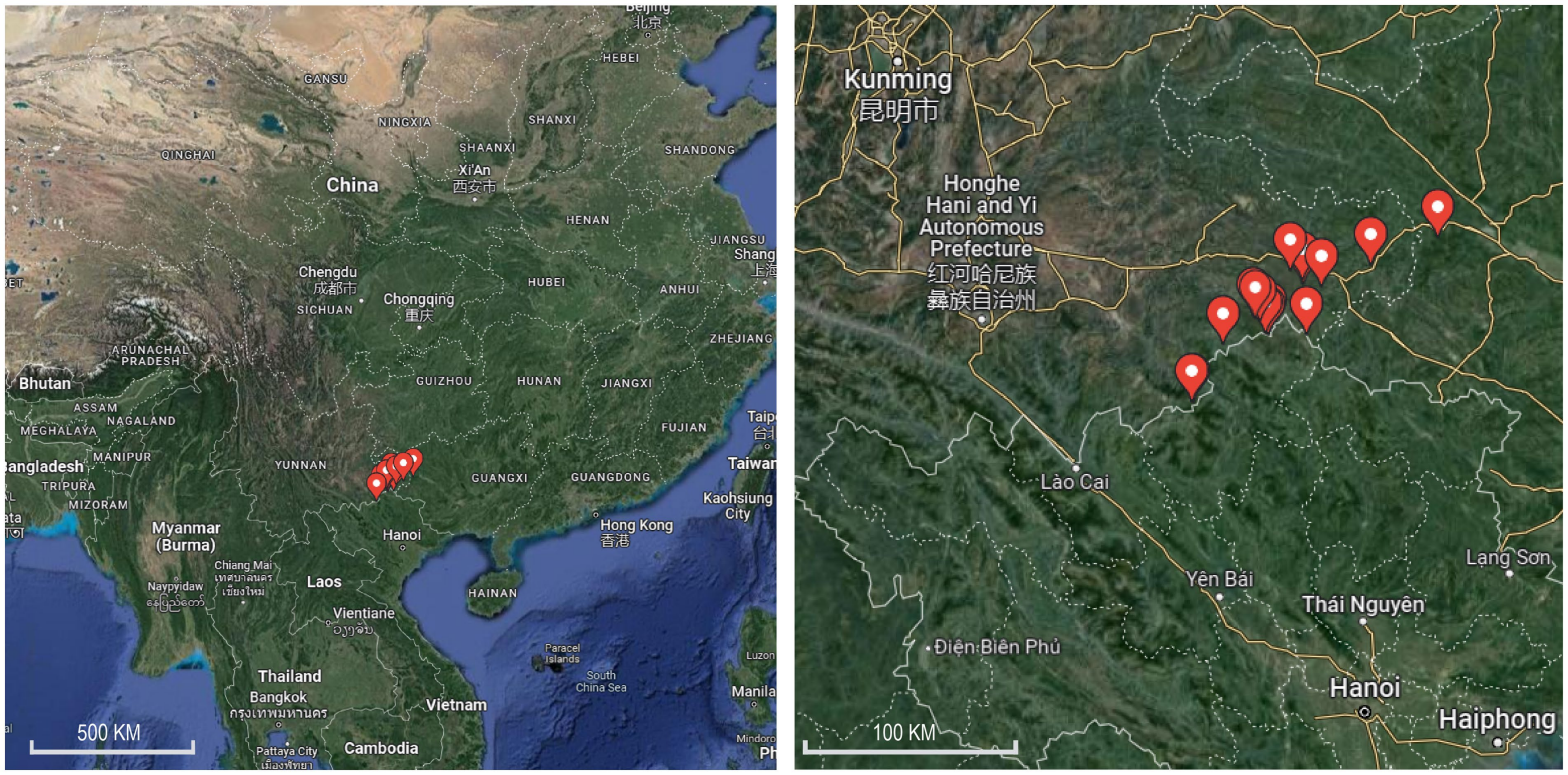
Map of mosquito collection sites along the Sino–Vietnam border in Yunnan Province, in 2020 and 2021.

From 15th July to 21st July 2021, blood-sucking mosquitoes were trapped in livestock corrals near the residential areas of Lida Township, Kuaiai Township, and Guichao Township in Funing County. The CDC Light Trap (John W. Hock Company) and an electric mosquito catcher were used at night and during the day, respectively. Mosquitoes were placed in a − 40°C freezer, and the frozen mosquitoes were removed and placed on ice for rapid mosquito sub-screening. Morphological classification of frozen adult mosquitoes and larvae mosquitoes were assigned using stereomicroscopy (Phenix Optical Technology Co., Ltd., Jiangxi, China). The identified mosquitoes were divided into freezing tubes with 1–50 according to the collection site and habitat and stored at −40°C. After the field survey, the frozen mosquitoes were stored in a freezer and transported to the laboratory for freezing at −80°C until examination. Information on the mosquito collection sites in the two counties is shown in Figure 1 and Supplementary Table S1.

### 2.2. Virus isolation and characterization

Virus isolation was performed using C6/36 Aedes albopictus cells. Mosquito homogenates were centrifuged at 4°C for 20 min at 12,000 rpm in a high-speed refrigerated centrifuge, and 40 μL of supernatant was aspirated and inoculated into a single layer of C6/36 cells in a 24-well plate and grown for 1 day (2 wells/sample). Two wells per plate of cell controls were set up and incubated at 32°C. Cytopathic effects (CPE) were observed and recorded continuously for 4–7 days. Isolates with obvious or suspected CPE were freeze-thawed once and transferred to 25-cm^2^ cell bottles for blind transmission to 3–6 generations. Viral isolates with regular CPE were freeze-thawed once and then aspirated into a freezing tube at −40°C for identification. During virus isolation, the group of C6/36 Aedes albopictus cell without mosquito supernatant was cultured as negative control.

Virus-specific RT-PCR was performed to identify the viral isolates. The viral nucleic acid was extracted using the ZYBIO Magnetic Beads Virus DNA/RNA Extraction Kit (ZYBIO, Chongqing, China) and the QIAamp® Viral RNA Mini Kit (Qiagen, Hilden, Germany) and reverse transcribed using M-MLV Reverse Transcriptase (Promega, Madison, WI, United States). Based on the literature, we synthesized PCR, detection, and Sanger sequencing primers (Supplementary Table S2) for a variety of arboviruses, such as flaviviruses, alphaviruses, and viruses in the family Reoviridae, and then designed primers from newly discovered viral sequences from Yunnan and Guangxi provinces published in recent years (GoTaq® Green Master Mix, Promega). Next, next-generation sequencing (NGS) was performed to detect viruses in the isolates with CEP, but negative for RT-PCR using the designed primers. A sequencing library was constructed using the NEBNext Ultra II Directional RNA Library Prep Kit (Illumina, San Diego, CA, United States). The library was subsequently sequenced on an Illumina NovaSeq 6000 (PE150) sequencing platform. Trimmomatic v0.36 (Bolger et al., 2014) was used to remove low-quality and short reads, and SPAdes v3.13.0 (Nurk et al., 2013) was used for metagenomic assembly. Blastn and Blastx were used to search for viral contigs. PCR detection and Sanger sequencing were performed using redesigned primers based on the viruses detected in samples with unknown viruses.

### 2.3. Phylogenetic analysis

The sequences obtained were aligned with related viral sequences using MAFFT v7.505 (Katoh and Standley, 2013) and phylogenetic trees were estimated for the aligned sequences using the maximum likelihood method available in IQtree v2.2.0 software with 1,000 bootstrap replications (Nguyen et al., 2015).

## 3. Results

### 3.1. Diversity and density of mosquito

A total of 39 species of mosquitoes from eight genera were collected from livestock pens, water containers, and village wood/bamboo forests in Malipo and Funing counties of Wenshan Prefecture, including 11 species of *Culex*, 12 species of *Anopheles*, five species of *Aedes*, five species of *Armigeres*, three species of *Ochlerotatus*, one species of *Toxorhynchites*, one species of *Malaya*, and one unclassified species of *Tripteroides*. From the corral, 10,512 mosquitoes from 30 species across five genera were collected, among which 71.79% (7,546/10,512), 18.54% (1,949/10,512), and 6.06% (637/10,512) of the total number of mosquitoes were *Culex tritaeniorhynchus*, *Anopheles sinensis*, and *Armigeres subalbatus*, respectively. A total of 412 mosquito larvae or pupae or both were collected from five townships in Malipo County, and 19 species belonging to seven genera of mosquitoes were taxonomically identified. A total of 237 mosquitoes of five species from three genera were trapped in wood and bamboo forests, with the main components of 223 *Aedes albopictus* mosquitoes. The mosquito species in different habitats are shown in Table 1.

**Table 1:** Mosquito species collected in different habitats.

Genus	Species	Habitat		
		Corrals	Containers of stagnant water	Woods/bamboo forests
*Culex*	*Culex tritaeniorhynchus*	+	−	−
	*Culex pipiens quinquefasciatu*	+	+	−
	*Culex bitaeniorhynchus*	+	−	−
	*Culex fuscocephala*	+	−	−
	*Culex halifaxia*	+	+	−
	*Culex pallidothorax*	+	+	−
	*Culex nigropunctatus*	+	−	−
	*Culex murrelli*	−	+	−
	*Culex jacksoni*	−	+	−
	*Culex mimulus*	−	+	−
	*Culex fuscanus*	+	+	−
*Anopheles*	*Anopheles sinensis*	+	−	−
	*Anopheles pseudowillmori*	+	−	−
	*Anopheles willmori*	+	−	−
	*Anopheles minimus*	+	−	−
	*Anopheles lindesayj*	+	−	−
	*Anopheles gigas baileyi*	+	−	−
	*Anopheles kochi*	+	−	−
	*Anopheles maculatus*	+	−	−
	*Anopheles nivipes*	+	−	−
	*Anopheles vagus*	+	−	−
	*Anopheles tessallatus*	+	−	−
	*Anopheles philippinensis*	+	−	−
*Aedes*	*Aedes albopictus*	+	+	+
	*Aedes annandalei*	+	+	+
	*Aedes desmotes*	+	−	+
	*Aedes vexans*	+	−	−
	*Aedes subalbopictus*	−	+	−
*Armigeres*	*Armigeres subalbatus*	+	+	+
	*Armigeres durhami*	+	+	−
	*Armigeres pallidofhorax*	+	−	−
	*Armigeres flavus*	−	+	−
	*Subgenus Leicesteria (Unclassified)*	−	+	−
*Ochlerotatus*	*Ochlerotatus albolateralis*	+	−	−
	*Ochlerotatus chrysolineatus*	+	+	+
	*Ochlerotatus elsiae*	+	+	−
*Toxorhynchites*	*Toxorhynchites gravelyi*	−	+	−
*Malaya*	*Malaya genurostris*	−	+	−
*Tripteroides*	*Subgenus Tripteroides (Unclassified)*	−	+	−

### 3.2. Isolation and molecular identification of mosquito-borne viruses

Of all the mosquitoes collected, 10,800 mosquitoes from 29 species were milled for virus isolation, including 10,472 mosquitoes collected from corrals, 232 mosquitoes trapped in wood and bamboo forests, and 96 feathered mosquitoes. We formed 537 mosquito pools based on mosquito species and collection sites. In total, 62 CPE virus-infected C6/36 viral isolates were isolated from seven mosquito species, namely *Culex trituberculatus*, *Anopheles sinensis*, *Aedes albopictus*, *Armigeres subalbatus*, *Armigeres durhami*, *Ochlerotatus albolateralis*, and *Culex pipiens quinquefasciatus*, of which 28 were isolated from *Culex trituberculatus* (Table 2). A total of 11 viruses were isolated and identified, including Banna virus (BAV) of the genus *Seadornavirus* and Yunnan orbivirus (YUOV) of the genus *Orbivirus* in the family Sedoreoviridae; Getah virus (GETV) of genus *Alphavirus* in the family Togaviridae; Tembusu virus (TMUV) of genus *Flavivirus* in the family Flaviviridae; Menghai rhabdovirus (MRV) of species *Almendravirus menghai*, genus *Almendravirus* in the family Rhabdoviridae; Nam Dinh virus (NDiV) of species *Alphamesonivirus 1* and Dak Nong virus (DKNV) of species *Alphamesonivirus 3*, classified into genus *Alphamesonivirus* in the family Mesoniviridae; unclassified Totivirus from the family Totiviridae, Sarawak virus (SWKV) from the family Alphatetraviridae; and Tanay virus (TANAV) and New Tanay virus (NTANAV) of the genus *Sandewavirus* from the newly proposed taxon Negevirus. Nine mosquito-borne viruses were identified by RT-PCR using specific virus primers, whereas the remaining two viruses, DNKV and SWKV, were identified for the first time by next-generation sequencing (NGS) and confirmed RT-PCR using redesigned primers. The number of each virus strain and its distribution among the mosquitoes are shown in Table 2.

**Table 2:** Isolation of viruses from collected mosquitoes.

Species/host	Total number	Groups	Virus	Virus identification										
				BAV	YUOV	TANAV	NTANAV	GETV	TMUV	MRV	NDiV	DKNV	SWKV	Toti virus
*Culex tritaeniorhynchus*	7,519	255	29	3	7	–	–	–	3	1	4	11	–	–
*Anopheles sinensis*	1936	87	19	11	–	–	–	–	–	–	1	6	–	1
*Armigeres subalbatus*	642	51	10	–	–	2#	5	–	–	–	2	1	–	–
*Aedes albopictus*	290	50	2	–	–	–	–	–	–	–	–	–	2&	–
*Armigeres durhami*	57	8	1	–	–	1	–	–	–	–	–	–	–	–
*Ochlerotatus albolateralis*	9	3	1	–	–	–	–	1	–	–	–	–	–	–
*Culex pipiens quinquefasciatu*	27	8	1	–	–	–	–	–	–	–	1	–	–	–
*Culex pallidothorax*	11	3	0	NA	NA	NA	NA	NA	NA	NA	NA	NA	NA	NA
*Culex fuscocephala*	14	4	0	NA	NA	NA	NA	NA	NA	NA	NA	NA	NA	NA
*Culex fuscanus*	13	2	0	NA	NA	NA	NA	NA	NA	NA	NA	NA	NA	NA
*Culex nigropunctatus*	1	1	0	NA	NA	NA	NA	NA	NA	NA	NA	NA	NA	NA
*Culex bitaeniorhynchus*	3	2	0	NA	NA	NA	NA	NA	NA	NA	NA	NA	NA	NA
*Culex mimeticus subgroup*	20	3	0	NA	NA	NA	NA	NA	NA	NA	NA	NA	NA	NA
*Culex halifaxia*	1	1	0	NA	NA	NA	NA	NA	NA	NA	NA	NA	NA	NA
*Anopheles vagus*	3	2	0	NA	NA	NA	NA	NA	NA	NA	NA	NA	NA	NA
*Anopheles tessallatus*	3	2	0	NA	NA	NA	NA	NA	NA	NA	NA	NA	NA	NA
*Anopheles minimus*	2	2	0	NA	NA	NA	NA	NA	NA	NA	NA	NA	NA	NA
*Anopheles gigas baileyi*	6	2	0	NA	NA	NA	NA	NA	NA	NA	NA	NA	NA	NA
*Anopheles maculatus*	4	2	0	NA	NA	NA	NA	NA	NA	NA	NA	NA	NA	NA
*Anopheles philippinensis*	4	2	0	NA	NA	NA	NA	NA	NA	NA	NA	NA	NA	NA
*Anopheles kochi*	1	1	0	NA	NA	NA	NA	NA	NA	NA	NA	NA	NA	NA
*Anopheles lindesayj*	4	1	0	NA	NA	NA	NA	NA	NA	NA	NA	NA	NA	NA
*Anopheles willmori*	13	3	0	NA	NA	NA	NA	NA	NA	NA	NA	NA	NA	NA
*Anopheles pseudowillmori*	3	2	0	NA	NA	NA	NA	NA	NA	NA	NA	NA	NA	NA
*Armigeres pallidofhorax*	3	3	0	NA	NA	NA	NA	NA	NA	NA	NA	NA	NA	NA
*Aedes annandalei*	1	1	0	NA	NA	NA	NA	NA	NA	NA	NA	NA	NA	NA
*Aedes vexans*	154	16	0	NA	NA	NA	NA	NA	NA	NA	NA	NA	NA	NA
*Aedes desmotes*	3	2	0	NA	NA	NA	NA	NA	NA	NA	NA	NA	NA	NA
*Aedes Unclassified*	14	8	0	NA	NA	NA	NA	NA	NA	NA	NA	NA	NA	NA
*Ochlerotatus elsiae*	36	7	0	NA	NA	NA	NA	NA	NA	NA	NA	NA	NA	NA
*Ochlerotatus Unclassified*	3	3	0	NA	NA	NA	NA	NA	NA	NA	NA	NA	NA	NA
In total	10,800	537	63	14	7	3	5	1	3	1	8	18	2	1

−, negative by RT-PCR with virus-specific primers; NA, not tested by RT-PCR; #, one isolate from a Armigeres subalbatus pool collected in the woods and another isolate from Armigeres subalbatus pool collected in a pig pen; &, two isolates from Aedes albopictus collected in a bamboo forest.

### 3.3. Viruses with a wide range of vertebrate or arthropod hosts

**Figure 2:**
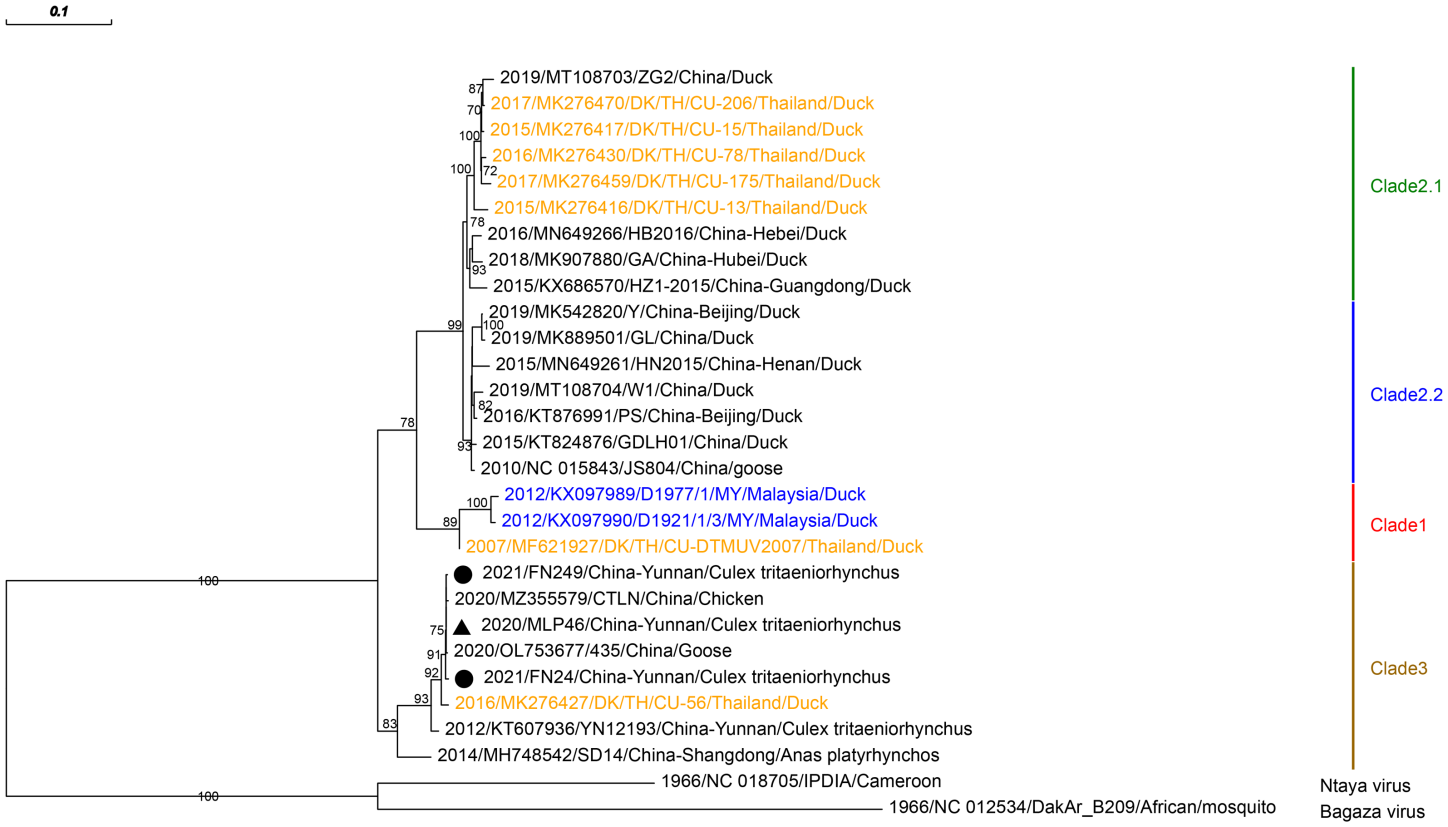
Phylogenetic analysis based on the nucleotide sequence of envelope protein sequences (1,503 bp) of TMUV. The phylogenetic tree was constructed by the maximum likelihood statistical method using IQtree v2.2.0 software. Bootstrap values were obtained from 1,000 replicates. Each TMUV sequence is denoted by year of isolation, GenBank accession number, strain, country of origin and isolation source. The sequences obtained from mosquitoes collected in Malipo county in this study are denoted by black triangle, and the sequences obtained from mosquitoes collected in Funing county in this study are denoted by black circle. Sequences from Malaysia and Thailand are indicated in blue and orange, respectively. Different clades are marked with different colors on the right. Branch scale bars are shown as 0.01 substitutions per site.

Three strains of TMUV were isolated and identified from three pools of *Culex tritaeniorhynchus*, captured in 2020 and 2021 in corrals of Malipo and Funing counties, respectively. We amplified the envelope protein of TMUV and reconstructed its phylogenetic relationships. Phylogenetic analysis revealed that the TMUVs identified in this study were in the newly identified clade 3. They clustered into a well-supported clade encompassing chicken TMUV (MZ355579), goose TMUV (OL753677), and duck TMUV (MK276427; Figure 2). The three TMUVs identified showed nucleotide identities ranging from 99.53 to 99.87% to the closest sequences of goose TMUV (OL753677).

**Figure 3:**
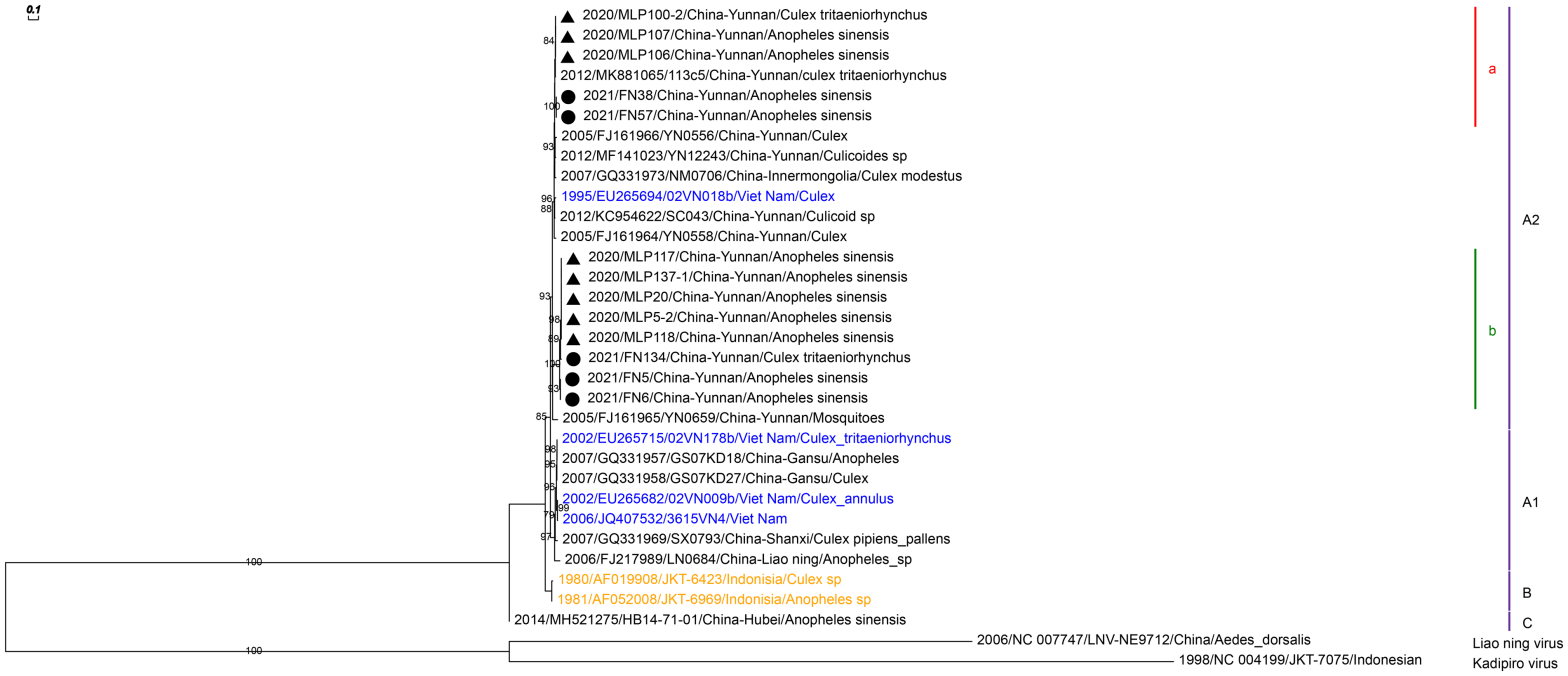
Phylogenetic analysis based on the nucleotide sequence of the partial 12th segment of BAV sequences (641 bp). The phylogenetic tree was constructed by the maximum likelihood statistical method using IQtree v2.2.0 software. Bootstrap values were obtained from 1,000 replicates. Each BAV sequence is denoted by year of isolation, GenBank accession number, strain, country of origin and isolation source. The sequences obtained from mosquitoes collected in Malipo county in this study are denoted by black triangle, and the sequences obtained from mosquitoes collected in Funing county in this study are denoted by black circle. Sequences from Vietnam and Indonesia are indicated in blue and orange, respectively. Different clades are marked with different colors on the right. Branch scale bars are shown as 0.01 substitutions per site.

We identified and sequenced five and eight strains of BAVs from mosquitoes in the corrals of Funing and Malipo counties, respectively. These viruses were isolated from two *Culex tritaeniorhynchus* pools and 11 *Anopheles sinensis* pools. 13 sequences of nearly complete dsRNA-binding protein gene in the 12th segment of the BAV were amplified, and the phylogeny was reconstructed based on this region. Phylogenetic analysis revealed that the BAVs clustered into two sister branches of the A2 genotype (Figure 3). BAVs in branch “a” showed nucleotide identities ranging from 98.05% to 99.67% with Yunnan BAV strain 113c5 (MK881065). Branch “b,” with 91.2% to 91.8% nucleotide identity to the closest sequence, forms a new well-supported branch.

**Figure 4:**
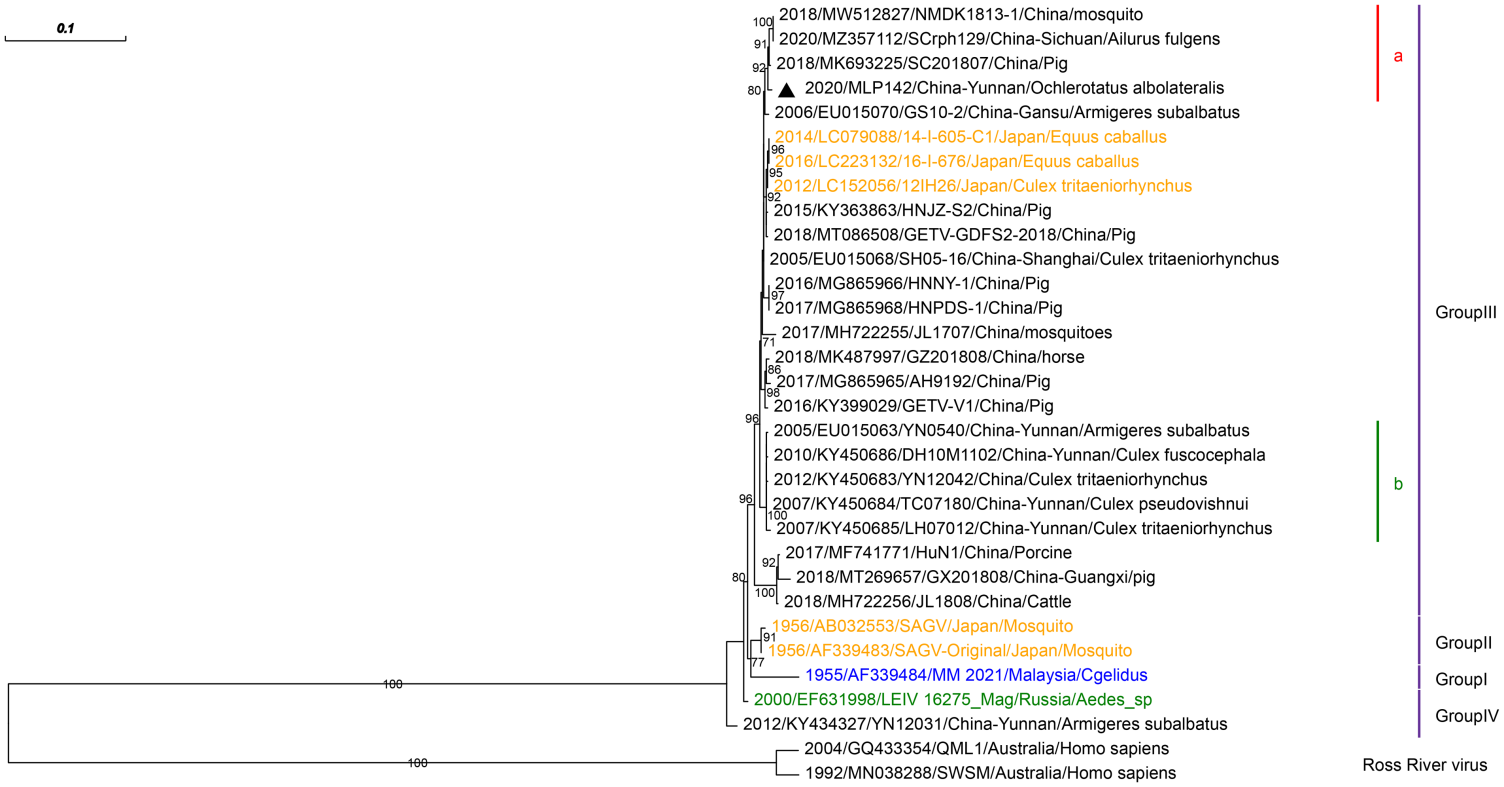
Phylogenetic analysis based on the nucleotide sequence of the *E2* gene sequences (948 bp) of GETV. The phylogenetic tree was constructed by the maximum likelihood statistical method using IQtree v2.2.0 software. Bootstrap values were obtained from 1,000 replicates. Each GETV sequence is denoted by year of isolation, GenBank accession number, strain, country of origin and isolation source. The sequences obtained from mosquitoes collected in Malipo county in this study are denoted by black triangle. Sequences from Malaysia, Japan and Russia are indicated in blue, orange and green, respectively. Different clades are marked with different colors on the right. Branch scale bars are shown as 0.01 substitutions per site.

We isolated only one strain of GETV from *Armigeres subalbatus* in the corrals of Malipo county. The partial envelope protein 2 gene of GETV isolated in this study was amplified, which was closest to that of GETV isolated from pigs in the Sichuan province of China (MK693225) with 99.59% nucleotide identity (Figure 4). We reconstructed the phylogeny based on the obtained sequences. Phylogenetic analysis showed that GETV group III clustered with GETVs isolated from China’s Sichuan and Gansu provinces.

In addition, YUOV was also tested in this study. Seven YUOV strains were isolated from *Culex tritaeniorhynchus* in the corrals of Malipo county. The sequences of the seven YUOV strains were genomically identical in the region tested, with the highest similarity to the 2018 Japanese cattle isolate ON-4/P/18 (LC585878), possessing a 97.91% nucleotide similarity.

### 3.4. Viruses restricted to insect hosts only

We isolated and identified six species of MSVs from mosquito pools: MRV, NDiV, DKNV, SWKV, TANAV, and NTANAV. In addition, a strain of Totivirus, which can infect either fungi or protozoa (Ghabrial and Nibert, 2009), was isolated in this study. DKNVs were isolated from 11 *Culex tritaeniorhynchuss* pools, six *Anopheles sinensis* pools, and one *Armigeres subalbatus* pool. The DKNVs showed 99.38% to 99.54% nucleic acid similarity to DKNV (NC_038297) found in Vietnam in 2007. SWKVs in this study were isolated from two *Aedes albopictus* pools collected in a bamboo forest and were closest to the Sarawak virus SWK-M26 (NC_040540) found in Malaysia, with 94.25% nucleic acid similarity. Other viruses, TANAV, NTANAV, MRV, Totivirus, and NDiV, which showed nucleotide identities ranging from 95.75% to 99.81% to viruses previously isolated in China, were isolated in our Sino–Vietnam border viral surveillance study. Three TANAVs, five NTANAVs, one MRV, eight NDiVs, and one Totivirus were isolated and distributed among six mosquito species (Table 2).

## 4. Discussion and conclusion

In our study, 39 species of mosquitoes from eight genera were collected near residential areas along the Sino–Vietnam border, mainly from livestock pens and nearby standing water containers and wood/bamboo forests. A total of 10,800 mosquitoes from 29 species were used for virus isolation. *Culex tritaeniorhynchus* accounted for 69.62% (7,519/10,800), and *Anopheles sinensis* accounted for 17.9% (1,936/10,800), and these species were the two most abundant species in this study. A total of 537 mosquito pools were mixed, and 62 viral isolates with CPE in C6/36 cells were isolated. Viruses from the isolates were classified into 11 categories based on the virus-specific primers used for RT-PCR. Each of the 61 viral isolates was identified with one viral category, whereas one viral isolate was identified with the co-existence of BAV and NDiV. We revealed the abundance of mosquito species, and the diversity of the pathogens they carry, in the vicinity of inhabited areas along the Sino–Vietnamese border.

Of the several important viruses that may become vertebrate pathogens, both TMUV and GETV were found initially in Malaysia and have been recently found in many places in China (Hamel et al., 2021; Li et al., 2022). TMUV can cause encephalitis and neurological disorders in birds and induce severe egg drop syndrome in ducks (Yu et al., 2022). TMUV was reported to cause epidemics affecting ducks in China in 2010, leading to huge economic losses and attracting widespread attention (Cao et al., 2011). A wide range of avian species, including geese, chickens, house sparrows, and pigeons, was confirmed to be naturally infected by TMUV (Tang et al., 2013a, 2015; Chen et al., 2014; Yan et al., 2017, 2022; Zhu et al., 2022), and infections in human have been demonstrated by serological tests or the presence of RNA (Wolfe et al., 2001; Tang et al., 2013b). Due to the lack of widespread surveillance, the virus is mostly reported in Thailand, Malaysia, and China, with few reports of TMUV in Vietnam (Hamel et al., 2021). The sequence of TMUVs we found at the Sino–Vietnam border was highly similar to that of avian TMUVs, and a possibility of the potential for local avian outbreaks cannot be ruled out. GETV is a mosquito-borne arbovirus, and GETVs have been shown to cause fever, rash, edema of the hind legs, and swelling of the lymph nodes in horses; GETVs cause miscarriages and other symptoms in pigs (Li et al., 2022). GETVs are widely distributed in the Asian continent and have been found in various animal specimens. These viruses have been isolated in 13 countries and have gradually spread from the tropics to temperate and even cold climate zones (Li et al., 2022). All GETVs are divided into four groups (GI–GIV) based on phylogenetic analysis of the *E2* gene sequence. The GIII group members have gradually become the most numerous isolates of GETV, and this group bears the most responsibility for causing animal diseases, with a widely distributed lineage and a trend of gradual expansion (Li et al., 2022). The GETV identified in this study was classified as GIII. TMUVs and GETV have both been isolated from mosquitoes in corrals. The presence of these viruses at the Sino–Vietnam border could pose a threat to the local poultry and pig farming industries.

BAV and YUOV were originally identified in the Yunnan region, and we confirmed that both viruses are still circulating in this region. BAV was first isolated from patients with encephalitis in Yunnan Province (Xu et al., 1990). and has been found in many locations in China, Vietnam (Nabeshima et al., 2008), Indonesia (Liu et al., 2016), and other areas. Compared with other known segmented double-stranded RNA viruses, BAV has a significantly higher nucleotide substitution rate, which gives BAV the capacity to adapt quickly to a new environment and host (Liu et al., 2016). Rearrangement events between different BAV genotypes have also been observed (Nabeshima et al., 2008). The BAVs we isolated were classified as subgroup A2, which comprises strains from South China and Vietnam (Xia et al., 2018). A previous study claimed that BAV strains from the same latitudes were almost 100% identical (Liu et al., 2016) yet we observed that two branches of BAVs, with approximately 8% nucleotide sequence divergence in the tested region, co-existed in both Malipo and Funing counties. Our findings extend the knowledge of the diversity of BAV distributions. YUOV was identified for the first time in the Yunnan province in *Culex tritaeniorhynchus* (Attoui et al., 2005), and YUOV has been isolated from vertebrates in Japan, Peru, and North America (Attoui et al., 2009; Murota et al., 2021; Viadanna et al., 2021). IgM and IgG against YUOV were detected in the serum of one patient at the China–Myanmar–Laos border in Yunnan Province, providing evidence of YUOV infection in the area (Wang et al., 2011). Our findings prove that YUOV is present in Wenshan Prefecture on the Sino–Vietnam border, a potential human pathogen that should be investigated in this area.

In addition, six MSVs and one Totivirus, which could produce CPEs in C6/36 cells, were identified. MSVs are commonly neglected as they cannot cause infections in humans and have almost no economic impact on the farming industry. The MSVs identified in this study accounted for approximately 60% (38/62) of the isolates. TANAV, NTANAV, MRV, Totivirus, and NDiV have been previously isolated in China and could be tested using primers designed to detect, which have no more than 5% sequence divergence to known viruses. The other two viruses, DKNV and SWKV, were identified for the first time in China using next-generation sequencing (NGS) and confirmed using RT-PCR. DKNV and SWKV were discovered in Vietnam and Malaysia, respectively (Kuwata et al., 2013; Sadeghi et al., 2017). Currently, DKNV and SWKV have only one sequence of genetic information available in GenBank. In addition to the location where they were originally found, we found these two viruses on the Sino–Vietnam border. This finding extends the current knowledge regarding the distribution of DKNV and SWKV. We speculate that these viruses may have a broader distribution in Asia.

We collected a large number of samples from multiple locations along the Sino–Vietnam border for virus isolation, followed by molecular identification of the isolates to determine the viral species, which is an accepted protocol for identifying the presence of viruses. However, the method still has limitations, as viruses that are less abundant or more difficult to isolate may be overlooked. For example, in another virus detection study on JEV, we amplified 1,500 bp of the E gene of JEV in raw samples rather than in culture supernatants. In addition, some isolable viruses may be overlooked due to experimental design and targeting issues. In this study, 32.26% (20/62) of isolates were not identified based on the primers we initially designed but were ultimately identified as DKNV or SWKV using RT-PCR with redesigned primers based on NGS data. Mosquito virome studies using large-scale metagenome sequencing have shown many advantages over the traditional virus identification methods (da Silva et al., 2021), providing an unbiased description of the viruses in the entire sample. However, this approach still has a notable limitation. Determining whether the viruses identified in NGS data can infect host cells or whether they are the main cause of any observed cellular CEP is difficult. We found the presence of Kaiowa and Guato viruses in our NGS data. However, we also identified them in the negative control cells using RT-PCR. Therefore, we defined them as viruses naturally carried by the host cells or those that did not cause cellular CEP, and they were not described in this study. We used NGS to identify viral isolates and reconfirmed the results using RT-PCR, which improved the accuracy of the assays to some extent. Viral surveillance and screening by multiple modalities (e.g., virus isolation, macro-genome sequencing, and antibody-level testing) are useful and essential for additional confirmation and early warning of emerging pathogens.

In summary, we revealed the abundance of mosquito species and the diversity of viruses they carry in the vicinity of inhabited areas along the Sino–Vietnamese border. Conducting a more extensive regional viral surveillance is necessary to reflect the changing epidemics of mosquito-borne viruses.

## Data availability statement

The data presented in the study are deposited in the GenBank repository, accession number OP675480-OP675514 and OP858811-OP858851.

## Author contributions

YTo and XG conceived and designed this study. FT and JH performed the experiments, analyzed the data, and prepared the initial draft of the manuscript. SS, ZC, YTa, ML, and CH contributed to sampling. All authors contributed to the article and approved the submitted version.

## Funding

This research was supported by the National Natural Science Foundation of China (grant no. 81560548), the National Key Research and Development Program of China (grant nos. 2018YFA0903000 and 2021YFC0863400), and the Key Project of Beijing University of Chemical Technology (no. XK1803-06).

## Conflict of interest

The authors declare that the research was conducted in the absence of any commercial or financial relationships that could be construed as a potential conflict of interest.

## Publisher’s note

All claims expressed in this article are solely those of the authors and do not necessarily represent those of their affiliated organizations, or those of the publisher, the editors and the reviewers. Any product that may be evaluated in this article, or claim that may be made by its manufacturer, is not guaranteed or endorsed by the publisher.
