# Supplementary material for: Survey of mosquito species and mosquito-borne viruses in residential areas along the Sino–Vietnam border in Yunnan Province in China
Source: Front Microbiol. 2023 Feb 23;14:1105786. doi: 10.3389/fmicb.2023.1105786 (PMC9996012; doi:10.3389/fmicb.2023.1105786)
Supplement: Supplementary file 1 [file Table_1.DOCX]

Table S1 Information of mosquito collection sites for pathogen detection along the Sino-Vietnam border in Yunnan Province, in 2020 and 2021.

| **Counties** | **Districts** | **Villages/Towns** | **East longitude** | **Latitude North** | **Altitude(m)** | **Habitat** | | |
| --- | --- | --- | --- | --- | --- | --- | --- | --- |
|  |  |  |  |  |  | **Corrals** | **Woods/bamboo forests** | **Containers of stagnant water** |
| Malipo | Mali | Xiananpu | 105°13'19" | 23°17'50" | 1126.7 | + | + | + |
|  |  | Nanduo | 105°12'49" | 23°16'24" | 1073.4 | + |  | + |
|  |  | Malipo | 105°11'41" | 23°17'01" | 1057 |  |  | + |
|  | Tianbao | Tianbaokouan | 105°26'16" | 23°34'38" | 107 |  |  | + |
|  |  | Balihe | 105°26'15" | 23°36'14" | 777 | + |  |  |
|  |  | Tianbao | 105°21'17" | 23°39'15" | 670.7 | + |  | + |
|  | Babu | Huangtian | 105°27'36" | 23°16'37" | 1024.1 | + | + | + |
|  |  | Bamen | 104°55'22" | 23°13'19" | 490 | + |  | + |
|  | Donggan | Baishagan | 105°07'48" | 23°22'40" | 1640 | + |  |  |
|  |  | Donggan | 105°09'37" | 23°21'35" | 1630 |  |  | + |
|  |  | Jinzhudang | 105°07'15" | 23°23'19" | 1535.3 | + |  | + |
|  | Mengdong | Mengdongyaozu | 104°43'03" | 22°52'45" | 1060 | + | + | + |
|  |  | Shangyangpo | 104°43'44" | 22°52'38" | 1060 | + | + | + |
| Funing | Lida | Wabeng | 105°33'22" | 23°33'35" | 1570 | + |  |  |
|  | Boai | Zhening | 106°18'07" | 23°50'57" | 468 | + |  |  |
|  | Guichao | Xiaomeng | 105°52'29" | 23°41'26" | 601 | + | + |  |

Note: “+ “ indicates implementation of mosquito collection.
