# Supplementary material for: Survey of mosquito species and mosquito-borne viruses in residential areas along the Sino–Vietnam border in Yunnan Province in China
Source: Front Microbiol. 2023 Feb 23;14:1105786. doi: 10.3389/fmicb.2023.1105786 (PMC9996012; doi:10.3389/fmicb.2023.1105786)
Supplement: Supplementary file 2 [file Table_2.DOCX]

Table S2 Primers designed for RT-PCR validation.

| Viruses | primer name | primer sequence(5'-3') | Target length（bp） | Target region | Reference |
| --- | --- | --- | --- | --- | --- |
| *alphavirus* | 6533f | TTGCAGGAGATACCAATGGA | 467 | RdRp | (Ledermann et al., 2014) |
|  | 6999c | AACATTCCGGATTTCATCAT |  |  |  |
| *flavivirus* | F1 | TACAACATGATGGGAAAGAGAGAGAA | 266 | NS5 | (Kuno et al., 1998) |
|  | F2 | GTGTCCCAGCCGGCGGTGTCATCAGC |  |  |  |
| BAV | 12-854-S | AAATTGATAGYGYTTGCGTAAGAG | 845 | Seg12 | (Xu et al., 2003) |
|  | 12-B2-R | GTTCTAAATTGGATACGGCGTGC |  |  |  |
| YUOV | YUOVSeg7S1 | AGCATTCGGTACGCAGTATCTCG | 470 | NS2 | (Attoui et al., 2005) |
|  | YUOVSeg7R1 | GCCGAGCCGATCATGTCACGTGT |  |  |  |
| NDiV | NDV-RDRP-F | TCGTGACGCTATTGTTTATC | 545 | RdRp | (Wang et al., 2016) |
|  | NDV-RDRP-R | CTGTGCGACTATTACCTGAA |  |  |  |
| Toti virus | OMRV5442F | ACCTACGCTACCAAGATGGCCA | 508 | RdRp |  |
|  | OMRV5949R | GATACGCCTCGATGTTGCTGGCT |  |  |  |
| GETV | GETV-F11 | TGCGCTATTCGAGGCACGAT | 1088 | E2–6K protein | (Li et al., 2017) |
|  | GETV-R11 | ATGATTATGGCAGCGAGCGG |  |  |  |
| NTANAV | newTanayF5049 | TGGTACAAGAAGTTGGCTCCTC | 1088 | RdRp | (Wang et al., 2018) |
|  | newTanayR6136 | ATATGAGTCGGAACGCCGAAG |  |  |  |
|  | newTanayF6069 | TCGCAACTACTTTTCGCTTTGG | 808 | RdRp |  |
|  | newTanayR6876 | TCGATCACATGGTCAGGGTC |  |  |  |
| TANAV | TanayRdRpF5807 | TGATTCCGTGCAGACTATCACT | 1033 | RdRp | MG673930.1 |
|  | TanayRdRpR6839 | TGGATGTGAGGGAGTTCGGT |  |  |  |
|  | TanayRdRpF4982 | ATTGACGCGCGACTTGTTTG | 996 | RdRp |  |
|  | TanayRdRpR5977 | GTGGACCAAACGTCGTAACA |  |  |  |
| MRV | MHrhabdovF8348 | CCAGAAACTGCATCATTTGGGT | 567 | RdRp | KX785335 |
|  | MHrhabdovR8914 | ACTGCTAGCTGGATAAGACGG |  |  |  |
| TMUV | TumbsuF34-1 | AGGCCGGGTAGTCAATATGC | 1154 |  | KT607936 |
|  | TumbsuR1187-1 | GCTGGAGCAATCAACATTAGCA |  |  |  |
|  | TumbsuF830-2 | TCATGCTAATGTTGATTGCTCCAG | 1174 |  |  |
|  | TumbsuR2003-2 | TCTCCGAATGGAGGTTCCAC |  |  |  |
|  | TumbsuF1232-3 | AAGCAGAAGGCAGGATTGTG | 1745 |  |  |
|  | TumbsuR2976-3 | GCTCTCTATCCAATAGCTCAGGTC |  |  |  |

Attoui, H., Jaafar, F.M., Belhouchet, M., Aldrovandi, N., Tao, S., Chen, B., et al. (2005). Yunnan orbivirus, a new orbivirus species isolated from Culex tritaeniorhynchus mosquitoes in China. *J Gen Virol* 86(Pt 12)**,** 3409-3417. doi: 10.1099/vir.0.81258-0.

Kuno, G., Chang, G.J., Tsuchiya, K.R., Karabatsos, N., and Cropp, C.B. (1998). Phylogeny of the genus Flavivirus. *J Virol* 72(1)**,** 73-83. doi: 10.1128/jvi.72.1.73-83.1998.

Ledermann, J.P., Zeidner, N., Borland, E.M., Mutebi, J.P., Lanciotti, R.S., Miller, B.R., et al. (2014). Sunguru virus: a novel virus in the family Rhabdoviridae isolated from a chicken in north-western Uganda. *J Gen Virol* 95(Pt 7)**,** 1436-1443. doi: 10.1099/vir.0.060764-0.

Li, Y.Y., Liu, H., Fu, S.H., Li, X.L., Guo, X.F., Li, M.H., et al. (2017). From discovery to spread: The evolution and phylogeny of Getah virus. *Infect Genet Evol* 55**,** 48-55. doi: 10.1016/j.meegid.2017.08.016.

Wang, J., Guo, X., Bao, J., Dong, X., Sun, X., Lin, Z., et al. (2016). Survey of mosquito vectors and their viral infections in Yunnan Chuxiong in 2014. *Chinese journal of Zoonoses* 32(06)**,** 581-588.

Wang, J., Wu, J., Li, N., Cao, Y., He, Y., Lin, J., et al. (2018). A new Tanay virus isolated from mosquitoes in Guangxi, China. *Arch Virol* 163(11)**,** 3177-3180. doi: 10.1007/s00705-018-3952-1.

Xu, L.H., Tao, S.J., Cao, Y.X., Wang, H.Q., Yang, D.R., He, Y., et al. (2003). [Genotyping of the Chinese isolates of coltivirus]. *Zhonghua Shi Yan He Lin Chuang Bing Du Xue Za Zhi* 17(4)**,** 346-350.
